# Nipah pseudovirus system enables evaluation of vaccines *in vitro* and *in vivo* using non-BSL-4 facilities

**DOI:** 10.1080/22221751.2019.1571871

**Published:** 2019-02-19

**Authors:** Jianhui Nie, Lin Liu, Qing Wang, Ruifeng Chen, Tingting Ning, Qiang Liu, Weijin Huang, Youchun Wang

**Affiliations:** Division of HIV/AIDS and Sexually Transmitted Virus Vaccines, National Institutes for Food and Drug Control (NIFDC), Beijing, People’s Republic of China

**Keywords:** Nipah, neutralization, pseudo virus, animal model

## Abstract

Because of its high infectivity in humans and the lack of effective vaccines, Nipah virus is classified as a category C agent and handling has to be performed under biosafety level 4 conditions in non-endemic countries, which has hindered the development of vaccines. Based on a highly efficient pseudovirus production system using a modified HIV backbone vector, a pseudovirus-based mouse model has been developed for evaluating the efficacy of Nipah vaccines in biosafety level 2 facilities. For the first time, the correlates of protection have been identified in a mouse model. The limited levels of neutralizing antibodies against immunogens fusion protein (F), glycoprotein (G), and combination of F and G (FG) were found to be 148, 275, and 115, respectively, in passive immunization. Relatively lower limited levels of protection of 52, and 170 were observed for immunogens F, and G, respectively, in an active immunization model. Although the minimal levels for protection of neutralizing antibody in passive immunization were slightly higher than those in active immunization, neutralizing antibody played a key role in protection against Nipah virus infection. The immunogens F and G provided similar protection, and the combination of these immunogens did not provide better outcomes. Either immunogen F or G would provide sufficient protection for Nipah vaccine. The Nipah pseudovirus mouse model, which does not involve highly pathogenic virus, has the potential to greatly facilitate the standardization and implementation of an assay to propel the development of NiV vaccines.

## Introduction

Nipah virus (NiV), a member of the newly defined *Henipavirus* genus of the Paramyxoviridae, was initially identified as the aetiological agent responsible for an outbreak of life-threatening encephalitis in individuals with close exposure to pigs in Malaysia and Singapore, where 276 respiratory or encephalitis cases were reported including 107 deaths [1]. Human-to-human transmission was subsequently observed in reemerging NiV outbreaks in Bangladesh and northeast India almost annually [2–4], which raised concerns of a possible widespread pandemic [5]. The recent outbreak of NiV encephalitis in India caused 18 confirmed infections, out of which 16 patients died [6]. The number of individuals at risk of NiV infection has reached more than 250 million in Bangladesh and the neighbouring regions of India. The total number of humans at risk of NiV infection might exceed two billion if all of the regions that have experienced NiV infection and in which *Pteropus* bats (the virus reservoir) reside naturally were included in the calculation [7]. The urgent need for research and development of antiviral products for NiV was listed among the priority diseases in the World Health Organization R&D Blueprint (http://www.who.int/csr/research-and-development/list_ofpathogens/en/).

Although no human vaccine for NiV has been approved, a variety of vaccine platforms have demonstrated the feasibility by employing one or two of the outer membrane proteins, fusion protein (F) and glycoprotein (G), as immunogens to induce protective immune responses, including various candidate vectored vaccines such as measles virus [8], rabies virus [9], vesicular stomatitis virus (VSV) [10], and canarypox virus [11]. A subunit vaccine employing a soluble glycoprotein (sG) from the related henipavirus Hendra virus (HeV), known as Equivac^®^HeV, has recently been approved to protect horses from HeV infection [12], which demonstrates the feasibility of NiV vaccine development. However, NiV is a highly pathogenic agent that should be handled in biosafety level 4 (BSL-4) facilities [13], which has limited the development of vaccines. No standardized measure has been established to predict the *in vivo* protection efficacy and correlates of protection for the immune response have not been fully defined, which has been another major barrier to developing candidate vaccines.

To avoid dealing with the infectious virus, several surrogate measures for antibody detection have been developed. ELISA assays and multiplexed microsphere assays were used to quantify the NiV-specific antibodies without discrimination of the neutralizing antibodies (NAbs) and non-NAbs [7]. Ephrin-B2 and ephrin-B3 have been identified as the cellular receptors for NiV and HeV [14–17]. Based on the Bio-Plex protein assay system, a high-throughput neutralization assay has been established to quantify NAb, which interferes with the interaction between ephrin-B2 (Nipah virus receptor) and soluble G [18]. However, this approach could not evaluate F-specific NAb. Pseudoviruses, displaying NiV-F and -G proteins on their particle surface, could largely mimic infectious NiV in the process of cell entry. The immune responses of candidate prophylactic vaccines targeting this process could be measured by the pseudovirus-based assay. An *in vitro* neutralization assay was developed based on two types of pseudovirus systems: vesicular stomatitis virus (VSV) and lentivirus-vectored pseudovirus. The VSV pseudovirus platform could generate high-titre pseudovirus but produced high background owing to the remaining recombinant VSV [19,20], while the lentivirus platform produced low-titre pseudovirus [21]. To our knowledge, pseudoviruses generated via these two systems could not have been utilized to develop *in vivo* animal infection models to provide an alternative to the infectious virus model for anti-viral evaluation.

We have developed a novel platform capable of generating high-titre pseudovirus with a modified HIV backbone vector and successfully established *in vitro* and *in vivo* infection models for a series of viruses, including rabies virus [22], Ebola virus [23], Marburg virus [24], Lassa virus [25], and Chikungunya virus [26]. In this communication, we developed pseudovirus-based neutralization assays for both the *in vitro* and *in vivo* analysis of immune responses stimulated by candidate NiV vaccines. The protective correlates for NAb were comprehensively investigated.

## Results

### Construction and characterization of pseudotyped virus with NiV F and G proteins

**Figure 1.**
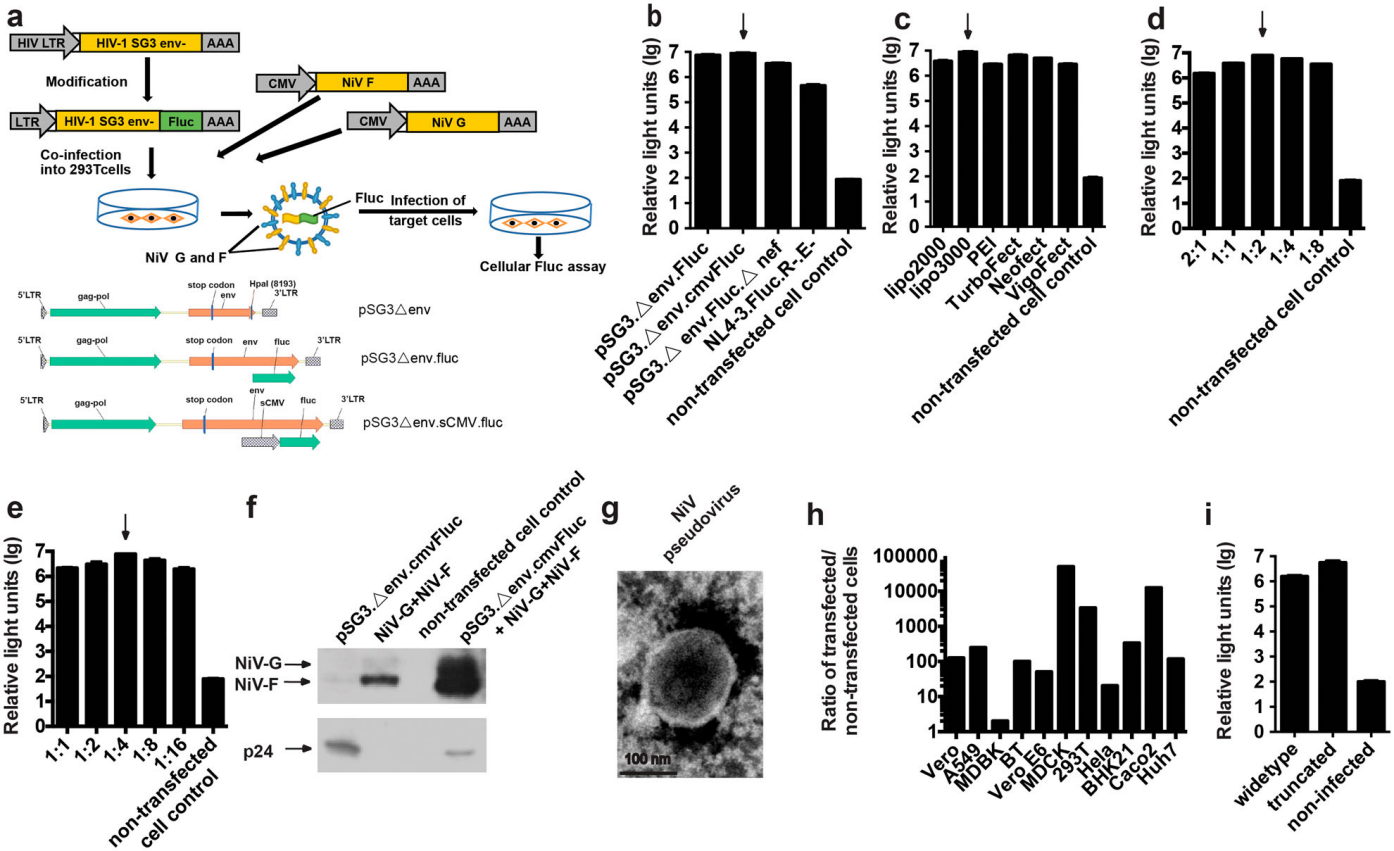
Optimization of the *in vitro* neutralization assay. (a) Schematic drawing of NiV pseudovirus production. Identification of the optimal backbone plasmid (b), transfection reagent (c), ratio of F and G plasmids (d), ratio of backbone and envelope plasmids (e), western blotting for F, G and p24 in pseudovirus (f), NiV pseudovirus under electronic microscopy (g), target cell (h). Non-transfected cells were included as negative controls in (b), (c), (d), (e) and (h). Culture supernatants from cells expressing different combinations of NiV proteins were filtered (0.45-μm pore size), then were pelleted through 25% sucrose cushion by ultracentrifugation at 100,000 g for 2.5 hr. The resulting viral pellets were resuspended in PBS and probed with FG immunized guinea pig serum and HIV-1 positive serum samples. As demonstrated by the highest luminescent signals, pSG3.Δenv.Fluc and Lipofectamine 3000 were determined to be the optimal backbone plasmid and transfection reagent, respectively. The optimal ratio for pcDNA3.1.F and pcDNA3.1.G was determined to be 1:2. The optimal ratio for the outer membrane protein genes and the backbone plasmids was found to be 1:4. (i) Effect of truncation of F and G on pseudovirus titre. Truncation of F and G could enhance titres of pseudovirus for about 3.6 fold. For Figure 1(b–e), h and i each experiment was performed twice (two replicates for each run) independently in different days. Mean with SD was shown for every condition.

To construct the NiV pseudovirus, the two outer membrane protein F and G genes were cloned into pcDNA3.1 to generate pcDNA3.1.F and pcDNA3.1.G, respectively (Figure 1(a)). Then, a series of backbone plasmids were tested with the F and G expressing plasmids to identify the optimal combination, as described previously [22–26]. (Figure 1(a)) Based on its high luminescent signals, pSG3.Δenv.cmvFluc was chosen for the following optimization (Figure 1(b)). The transfection reagents were compared to identify the optimal reagents to yield high-titre virus. For NiV pseudovirus generation, Lipofectamine 3000 achieved the highest titre (Figure 1(c)). Subsequently, the optimal ratio for pcDNA3.1.F and pcDNA3.1.G was determined to be 1:2 (Figure 1(d)). Similarly, the ideal ratio for the outer membrane protein genes and the backbone plasmids was found to be 1:4 (Figure 1(e)).

To verify the incorporation of proteins by NiV pseudovirus, the surface proteins of the particle were investigated using western blotting. Compared with the mock pseudovirus generated by transfection of 293 T cells with pSG3.Δenv.cmvFluc only, the F and G proteins were shown to have been assembled into the NiV pseudovirus (Figure 1(f)). When the NiV pseudoviruses were investigated using electron microscopy, most of the particles showed the typical morphology of HIV with a diameter of about 120 nm (Figure 1(g)), owing to the HIV capsid in the core of the pseudovirus. However, when the NiV pseudovirus was used to infect a number of mammalian cells, a wide cellular tropism was observed with all of the employed cells able to be infected by the pseudovirus efficiently, which is a characteristic of NiV rather than HIV. Among the potential target cells, the highest relative light unit (RLU) values were observed when MDCK cells were infected (Figure 1(h)). Thus, the *in vitro* neutralization of NiV pseudovirus was established based on MDCK cells.

It is reported that truncation of F and G could enhanced the pseudovirus titres [27]. When we tested the truncated and widetype F and G for pseudovirus generation, 3.6 fold enhancements of titres were observed due to truncation as described previously [27]. (Figure 1(i)).

### Development of the *in vivo* NiV pseudovirus infection model

**Figure 2.**
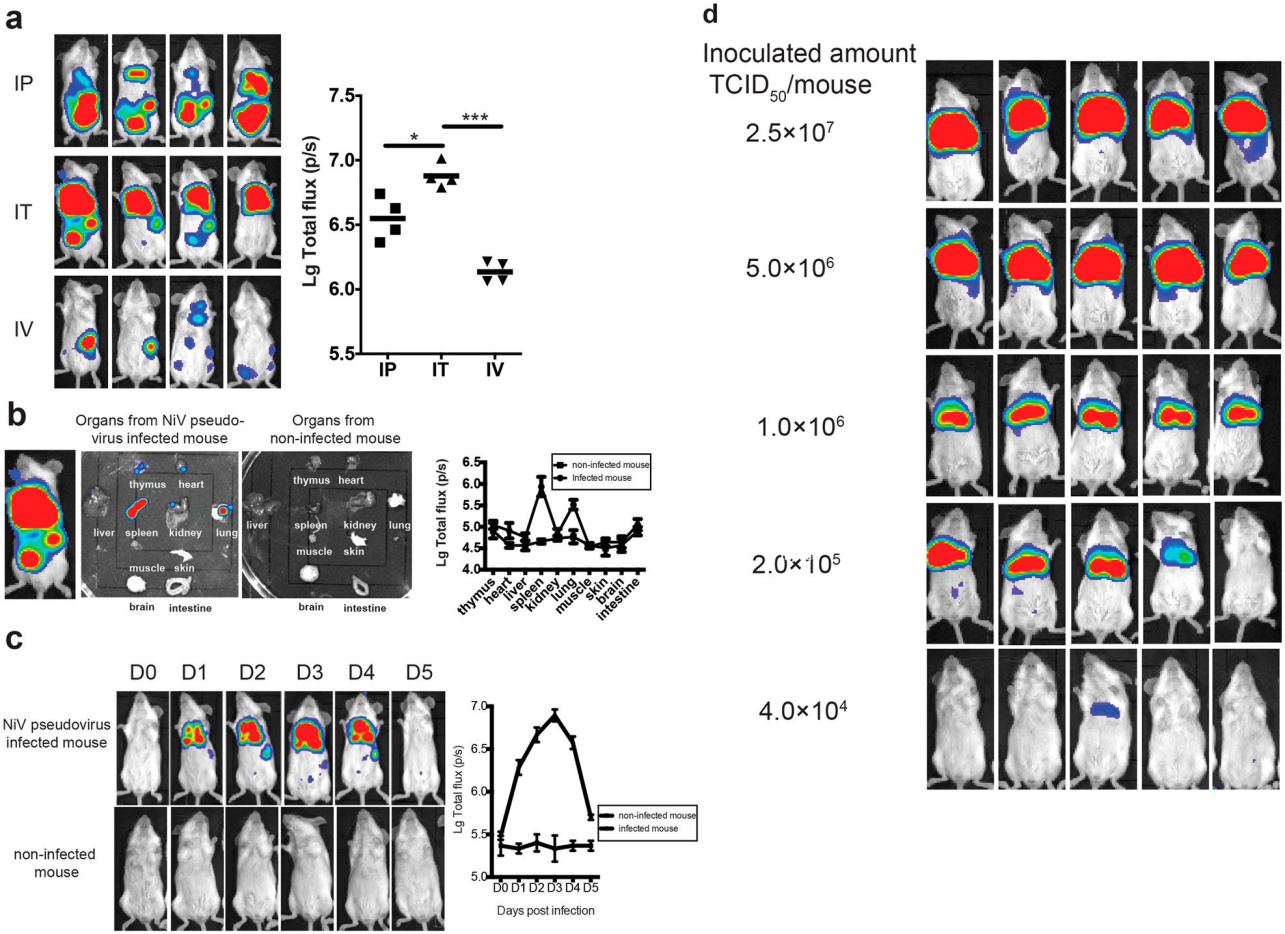
Development of *in vivo* imaging mouse model for NiV pseudovirus. (a) Optimization of the infection routes. The mice were inoculated with NiV pseudovirus (2.5 × 10^7^ TCID_50_) and the luminescent signals were detected at 2 dpi. Three challenge routes were investigated, including intraperitoneal (IP), intrathoracic (IC), and intravenous (IV) injections; the IT route yielded the highest signals compared with the other two routes. (b) Biodistribution of the NiV pseudovirus in mice. Various organs of the mice challenged via the IT route (in Figure 2(A)) were investigated using the luminescence detector. The total flux for each organ was collected from three mice, and the images of organs from one mouse are presented. Organs from non-infected mouse were included as negative control. High levels of flux were observed in the spleen and lung, the cast of which could also be found on the surface of the live mouse. (c) Identification of the optimal time point for detection. Flux signals for each mouse (3 mice in total) following inoculation with 4 × 10^6^ TCID_50_ were recorded every 24 h for up to 5 days. The optimal detection point was determined as 3 dpi, which showed the highest signal level. Non-infected mouse were included as negative control. (d) Determination of the animal infectious dose. Fivefold serially diluted NiV pseudoviruses were injected IT into five groups of mice (5 mice/group) at an initial dose of 2.5 × 10^7^ TCID_50_. The 50% animal infectious dose (AID_50_) for the pseudovirus was calculated to be 8.8 × 10^4^ TCID_50_, and the pseudovirus dose was determined to be 50 AID_50_ for the *in vivo* infection assay, which is equivalent to 4.4 × 10^6^ TCID_50._

To investigate whether the NiV pseudovirus preparations could be used to establish an *in vivo* infection model, we inoculated Balb/c mice with pseudotyped virus through a variety of routes including: intraperitoneal (IP), intrathoracic (IT), and intravenous (IV) injections; the IT route turned out to yield the highest signals compared with the other two routes (*p* < .05, Figure 2(a)). Various organs of a mouse challenged via the IT route were investigated using the luminescence detector. High levels of flux were observed in the spleen and lung, the cast of which could also be found on the surface of the live mouse (Figure 2(b)).

We next determined the optimal time point for luminescence detection in mice following IT injection of the NiV pseudovirus. To achieve this, the flux signals for each mouse (3 mice in total) were recorded every 24 h after the inoculation, for up to 5 days. As shown in Figure 2(c), the signals increased steadily and reached the highest level by day 3 post-infection. Then, the signals began to decline sharply from day 4 and were barely detectable by day 5. The optimal detection point was then determined as 3 days post-inoculation (dpi) based on these findings.

Finally, we determined the optimal amount for pseudovirus injection. To this end, 5-fold serially diluted NiV pseudoviruses were injected IT into five groups of mice (5 mice/group) with an initial dose of 2.5 × 10^7^ 50% tissue culture infective dose (TCID_50_) as predetermined by an *in vitro* infection assay (Figure 2(d)). The 50% animal infectious dose (AID_50_) for the pseudovirus was calculated to be 8.8 × 10^4^ TCID_50_. To make sure all the challenged mice presenting detectable and uniform signals, the pseudovirus dose was determined to be 50 AID_50_ for the *in vivo* infection assay, which is equivalent to 4.4 × 10^6^ TCID_50_.

### Correlation between the protective efficacy and NAbs in the passive immunization model

To characterize the protective efficacy of the NAbs, three groups of guinea pigs (2 animals/group) were immunized with plasmids expressing NiV F, G, and both, respectively. Three types of sera were obtained with NAbs specifically targeting F, G, and both F and G. The geometric means of the 50% inhibitory dilution (ID_50_) values were determined as 13,032, 15,668, and 20,184 for the F, G, and FG groups using the *in vitro* NiV pseudovirus neutralization assay (data not shown). The inhibition of the NiV pseudovirus by F and G immunized sera could prove in another way the presence of the F and G in the pseudovirus.

To investigate the protective efficacy of transfused F, G, and FG anti-sera, the sera from immunized guinea pigs were first diluted to the same predefined potency with an ID_50_value of 10,000, which was designated as level 1. Subsequently, the other three levels were acquired by 3-fold serial dilutions. Mice were randomly assigned to four groups (6 mice/group) and received injections of the four types of immunized sera with the corresponding dilution. One hour after passive transfusion, each mouse was bled for serum collection. Then, the mice were inoculated with the NiV pseudovirus and luminescent signals were detected at 3 dpi.

**Figure 3.**
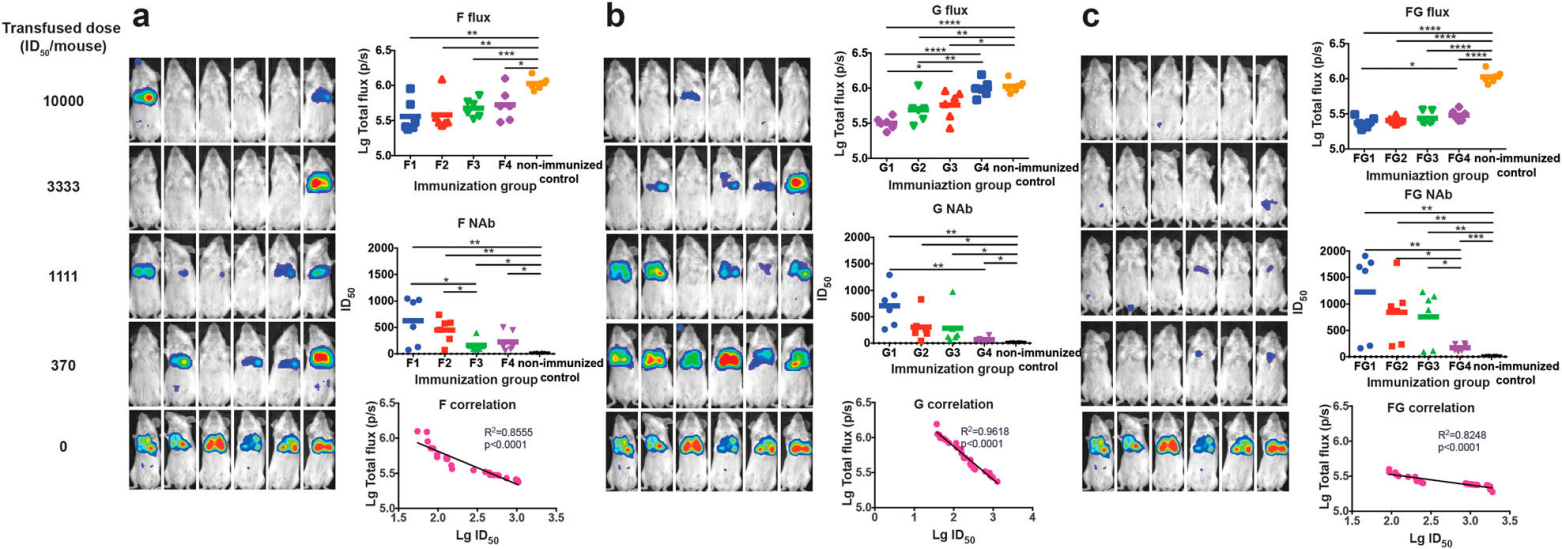
Identification of the protective correlates for passive immunization. Four groups of Balb/c mice (6 mice/group) were passively inoculated with 3-fold serially diluted anti-sera for immunogens F (a), G (b), and F and G combined (c), respectively. One hour after passive transfusion, each mouse was bled for serum collection. Then, the mice were inoculated with the NiV pseudovirus and the luminescent signals were detected at 3 dpi. Flux signals and NAb titres of the serum samples were detected for each mouse. Mice transfused with sera from non-immunized guinea pigs were included as control. The flux signals and NAb titres of each group were compared with the control group using student’s t test (*p < .05, **p < .01, ***p < .001, ****p < .0001). The correlation of the log-transformed values for the flux and NAb titres were analysed for each immunogen. Good linear correlations were found between the log-transformed total flux and the ID_50_ values for each immunogen. The limited full protection levels of NAbs were identified as 148, 275, and 115 for immunogens F, G, and FG, respectively. For Figure 3(a–c), all the three experiments were performed simultaneously with the same pseudovirus control.

For the F passive immunization, both the infected mice ratio and the average flux density, representing the amount of infected pseudovirus, were found to increase with the decreasing amount of transfused neutralizing sera (Figure 3(a)). Furthermore, NAbs were found in the mouse serum samples collected just before virus challenge, which also showed a dose-dependent effect (Figure 3(A)). When we associated the flux density of the bioluminescence with the NAbs, a good linear correlation was found between the log-transformed total flux and the ID_50_ values (*R*^2^ = 0.8555, *p* < .0001). The mice were found to be completely protected when the NAb level reached 148 or higher for the F immunogen.

As shown in Figure 3(b,c), similar dose-responses were also observed in the G and FG groups. When we checked the Nab titres individually in the breakthrough mice, limited protection levels were identified, 275 and 115, for immunogens G and FG, respectively.

### Protective efficacy of the DNA vaccines evaluated in the pseudovirus *in vivo* infection model

**Figure 4.**
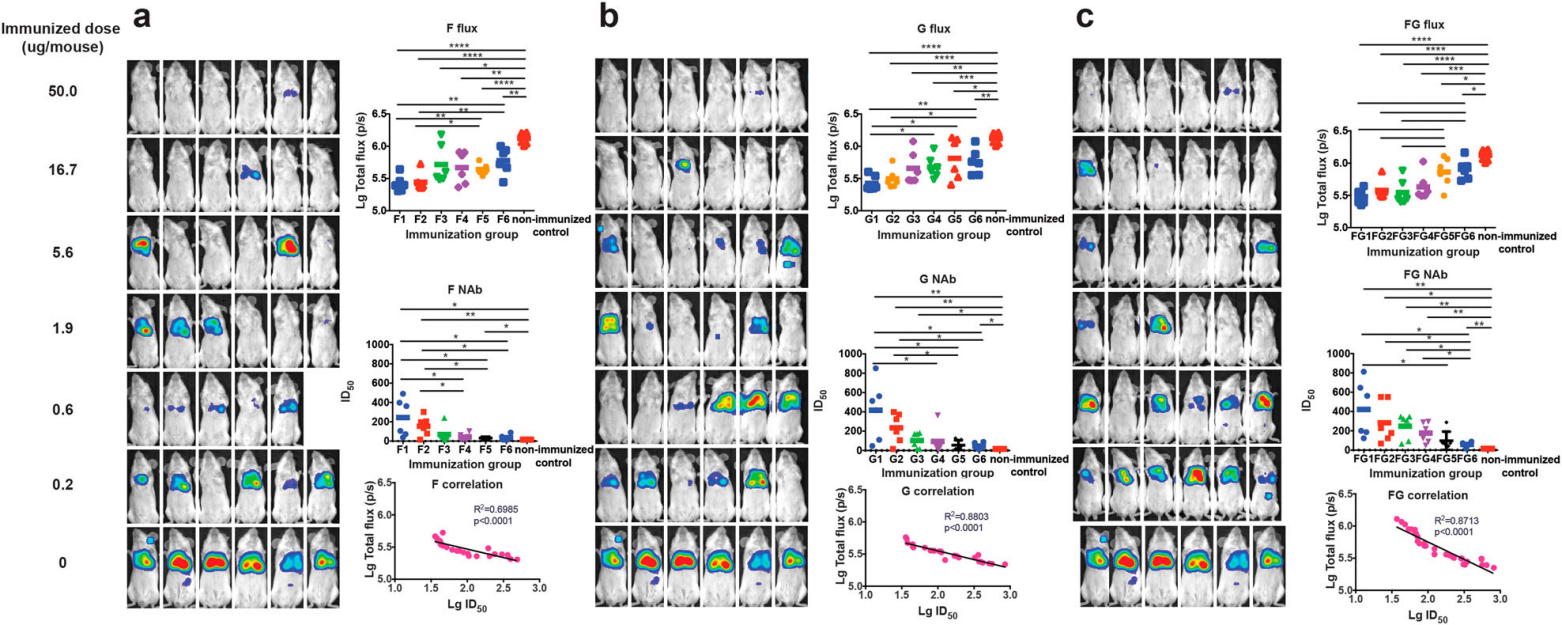
Identification of the protective correlates for active immunization. (a) For F antigen, six groups of mice (6 mice/group) were actively inoculated with serially diluted DNA vaccines (3-fold serial dilutions with an initial amount of 50 µg/mouse) for immunogens F (a), G (b), and F and G combined (c), respectively. Fourteen dpi, mice were bled for serum collection and challenged with the NiV pseudovirus. Luminescent signals were detected 3 days after infection. The flux signals and NAb titres were detected for each mouse. Mice with non-immunization were included as control. The flux signals and NAb titres of each group were compared with the control group using student’s t test (*p < .05, **p < .01, ***p < .001, ****p < .0001). The median effective dose (ED_50_) values were determined to be 3.79 μg (95% confidence interval: 1.13–13.13 μg), 4.00 μg (95% CI: 0.82–28.71 μg), and 3.38 μg (95% CI: 0.88–12.00 μg) for the DNA vaccines expressing F, G, and FG, respectively. The correlation between the log-transformed values for the flux and NAb titres were analysed. Significant linear correlations were found between the log-transformed total flux and the ID_50_ values for each immunogen. The limited protection levels of NAbs were identified as 52, 170, and 123 for immunogens F, G, and FG, respectively. For the F immunogen study, one mouse in the fifth group died after anesthetization just before bioluminescence detection, so the data for this animal were not included in the analysis. Data for Figure 4(a–c) were generated in one big experimental set-up.

In this section, we aimed to investigate the potential protective potency of the immunogens F, G, and both F and G. Towards this end, we serially diluted the DNA vaccines (3-fold serial dilution with an initial amount of 50 µg/mouse) and injected mice intramuscularly followed by electroporation. Fourteen dpi, mice were bled for serum collection and challenged with the NiV pseudovirus. For the F immunogen study, one mouse in the fifth group (0.6 µg/mouse) died after anesthetization just before bioluminescence detection, so the data for this animal were not included in the analysis. As shown in Figure 4(a), obvious dose-responses were found for the pseudovirus in the *in vivo* protection assay; the median effective dose (ED_50_) value was determined as 3.79 μg (95% confidence interval: 1.13–13.13 μg) for the DNA vaccine expressing F protein. NAb levels were found to be inversely proportional to the total flux intensity; a linear correlation was found between the log-transformed total flux and the ID_50_ values (*R*^2^ = 0.6985, *p* < .0001). It was observed that the mouse could be fully protected when the NAb level reached 52 or higher for the F DNA vaccine active immunization.

As shown in Figure 4(b,c), similar phenomena were observed in the G and FG groups. For the DNA vaccine immunization, the ED_50_ values for G and FG were determined to be 4.00 μg (95% CI: 0.82–28.71 μg) and 3.38 μg (95% CI: 0.88–12.00 μg), respectively. The limited full protection levels of NAbs were identified as 170 and 123 for immunogens G and FG, respectively. Collectively, these findings demonstrated that the pseudovirus *in vivo* assay could be employed to evaluate the effectiveness of vaccines targeting the outer membrane proteins of NiV.

## Discussion

To date, no clinical trials have been initialized for candidate NiV vaccines. The sporadic outbreaks of NiV make it almost impossible to carry out large-scale phase III clinical trials to investigate the efficacy to support vaccine licensure. Identification of the minimal levels of immune responses for protection might offer satisfactory evidence for regulatory approval; although accurate correlates for protection have not been completely characterized partially owing to the lack of standardized approaches for the investigation of immune correlates. Evaluation assays employing infectious NiV have to be carried out in BSL-4 biocontainment facilities, making the large-scale testing of samples more time-consuming. The handling of such infectious viruses is labour-intensive and monitoring the status of challenged animals for the *in vivo* infectious virus assay is time-consuming, requiring at least two weeks [28–32]. Clearly, developing alternative measures that are applicable in most basic and clinical laboratories would facilitate the evaluation of candidate NiV vaccines, and the identification of correlates of protection.

Most of the prophylactic NiV vaccine candidates were focused on the NiV–F and/or –G outer membrane proteins [8–11]. NiV pseudovirus, presenting native F and G proteins on the surface, could be used to detect NAbs, which interfere with the process of virus attachment and fusion. In addition, the pseudovirus assays could be potentially utilized to investigate other functional antibodies, such as antibody-dependent cellular cytotoxicity-inducing antibodies based on our experience with Ebola virus [23]. Two types of pseudovirus system platforms, VSV [19,20] and lentivirus [21], have been reported to generate NiV pseudovirus to quantify the Nabs *in vitro*. The pseudoviruses based on VSV were generated by transfecting the producer cell with heterologous envelope protein genes followed by infection with recombinant VSV, lacking the VSV-G gene in its genome [33]. Although the deficient VSV genome does not contain the G gene, recombinant VSV displays VSV-G on the virus surface. When VSV infects the pseudovirus producer cell, VSV-G anchors in the cell membrane, which can then be incorporated into part of the pseudovirus membrane during the process of budding to generate chimeric and/or pure VSV pseudovirus together with the expected pseudovirus with heterologous envelope proteins. Because a broad spectrum of cell types can be efficiently infected with VSV, the chimeric and pure VSV pseudoviruses would provide a false result indicating that the pseudovirus could not be completely neutralized [19,20]. The lentivirus-based pseudovirus system was not employed for NiV pseudotypes until the truncated F gene was introduced [21]. And the introduction of both truncated F and G could further improve the titres of NiV pseuovirus [27]. Although the pseudovirus with the truncated F and the G genes could efficiently infect target cells *in vitro*, the unexpected effects of the truncated F and G should be considered when this type of pseudovirus is used for vaccine evaluation, especially for vaccines employing full-length F and G. Through modification of the backbone plasmids, we generated pseudoviruses with full-length F and G, similar to the infectious virus, at even higher titres (10^8^ TCID_50_/ml, 100-fold higher than that previously reported [21]) without concentration, which could meet the requirements for *in vitro* and *in vivo* neutralization assays. The crude pseudovirus was used in most of assays, which was just purified via a 100 kDa ultrafiltration. When we compared the pseudoviruses of treated and untreated by ultracentrifugation, the ultracentrifugation-treated material had 50% greater sensitivity (data not shown), which might due to the partial removal of F or G trimers/tetramers.

For *in vivo* NiV pseudovirus infection, IT injection was identified as the optimal route of inoculation to generate high and homogenous flux signals in mice. This approach ensures the pseudovirus reaches the lung tissue easily, as this tissue serves as the major target for the infectious virus in mice [32,34,35]. Besides lung tissue, the spleen acted as another major virus reservoir, similar to the wild-type virus model [34]. Different types of target cells in the two organs are infected: macrophages and multinucleated giant cells play a key role in NiV infection in the spleen, and bronchial epithelial cells and alveolar macrophages serve as the major target in lung tissue [21]. A number of pseudotyped viruses used the liver as a reservoir, such as rabies virus [22], Ebola virus [23], Marburg virus [24], and Lassa virus [25]. Although replication of infectious NiV could be detected in the liver in some mice challenged with the infectious virus [36], the NiV pseudovirus bypassed the liver sink, showing the same features as another previously reported pseudovirus in a mouse model [21]. Collectively, although no clinical signs were observed, the NiV pseudovirus could mimic the infectious characteristics of wild-type virus in the mouse model and offer an alternative approach to investigate the correlates of protection for vaccines targeting attachment and entry.

Three patterns of DNA vaccine (F, G, and a mixture of F and G) could effectively elicit high-titre NAbs (13032-20184) in guinea pigs when three immunizations with two-week intervals were employed through intramuscular injection followed by electroporation. The NAb titres of the guinea pig sera were comparable to the highest previously described titres [7,8,10,11,31,37–42]. When F and G were compared, G induced relatively higher titres than F, consistent with previous reports [37,40,43]. Even with single immunization in mice, potent NAbs could be induced and offer protection from pseudovirus infection through DNA vaccination with electroporation. The NAb titres could reach 5–10-fold higher than the minimal protective level in mice. This suggested that DNA injection followed by electroporation could serve as an alternative immunization strategy for NiV vaccine candidates.

For Hendra virus, which is most similar to NiV, a NAb titre as low as 32 was identified as the minimal level for complete protection in cat and ferret models following infectious virus challenge [44]. However, no such correlates were determined for NiV candidate vaccines. In this communication, we investigated the correlates of protection for the two major immunogens: F and G, and a mixture of both (FG) in passive and active immunizations. For passive anti-serum transfer analysis, the reduction in flux signals, representing the amount of infectious pseudovirus *in vivo*, showed a stringently inverse correlation with the NAb titres for all immunogens, which confirmed the protective role of NAb irrespective of the type of immunogen employed. It is believed that NAbs might act as the main mediators of protection for NiV vaccine candidates, as evidenced by the protective efficacy of passive antibody transfer in infectious virus animal models [7]. When the anti-sera combination of G and F was inoculated into mice before challenge, higher efficiency of protection was observed compared with the single serum transfer, which might suggest the synergistic effect of the two outer membrane proteins on protection, but these differences were not statistically significant. Immunization with F, G, or FG could actively elicit high-titre NAbs in mice. However, no significant difference was found in the ED_50_ values among these inoculation patterns. Although the NAbs titres induced by F were slightly lower than those induced by G and FG, F could provide similar full protection at relatively low titres *in vivo*. So for the three types of immunization patterns for the DNA vaccines, the same amount of immunogen could induce similar protection *in vivo*. When the passive and active immunizations were compared, the minimal NAb levels for active vaccination were relatively lower than those for passive antibody transfer. This suggested that other types of functional antibodies or cellular immune responses might play a role in protection during active vaccination. Although correlates of protection for F, G, and FG were identified, how these data can be applied to the infectious virus model and ultimately to vaccine efficacy in humans will be an important focus of future work.

In short, both pseudovirus-based neutralization assays *in vitro* and *in vivo* have some specific advantages. The absence of highly pathogenic virus in the experimental procedure greatly facilitates the implementation of the assay, thereby potentially propelling the development of NiV vaccines and therapeutics; in addition, the pseudovirus *in vivo* assay is less labour-intensive and requires a shortened experimental time (from 2 weeks to just 3 days) compared with the traditional assay [28,32,45]. The Nipah pseudovirus assay also inherits all of the advantages of recombinant virus assays, namely high throughput, high reproducibility, and high versatility of the virus strains.

## Materials and methods

### Cells

Vero (American Type Culture Collection [ATCC], CCL-81), Vero E6 (ATCC, CRL-1586), MDBK (ATCC, CCL-22), MDCK (ATCC, CCL-34), BHK21 (ATCC, CCL-10), 293T (ATCC, CRL-3216), BT (ATCC, CRL-1390), A549 (ATCC, CRL-185), HeLa (ATCC, CCL-2), Caco-2 (ATCC, HTB-37), and Huh-7 (Japanese Collection of Research Bioresources [JCRB], 0403) cells were maintained in high glucose DMEM (GIBCO) supplemented with 10% FBS (GIBCO), penicillin (100 IU/ml), streptomycin (100 μg/ml), and HEPES (20 mM) in a 5% CO_2_ environment at 37 °C and passaged every 2–3 days.

### Construction of the Nipah envelope-expressing plasmids

For pseudoviruses construction, humanized F and G genes from a Malaysian strain (GenBank: AF212302) were cloned into mammal expression plasmid pcDNA3.1 to generate the envelope plasmids pcDNA3.1.F and pcDNA3.1.G, respectively. For DNA vaccination, the same two genes were inserted into another expression plasmid pDRV1.0 (kindly provided by Yiming Shao, China CDC) with the full-length CMV promoter, using restriction endonuclease digestion (*Bam*HI and *Xho*I) and direct ligation (In-Fusion) according to the manufacturer’s instructions (Clontech).

### Production and titration of pseudoviruses

NiV pseudoviruses were generated and titrated using methods similar to rabies pseudovirus, as described previously [22]. Briefly, mammalian cells were cotransfected with NiV envelope protein G and F expressing plasmids and the HIV packaging vector using different types of transfection reagents following the manufacturer’s instructions, including Lipofectamine 2000 (Invitrogen, 11668019), Lipofectamine 3000 (Invitrogen, L3000015), VigoFect (Vigrous Biotechnology, T001), TurboFect (Thermo Scientific, R0531), Neofect (Neofectbiotech Co), or PEI (Alfa Aesar, 43896). Forty-eight hours post transfection, NiV pseudovirus-containing culture supernatants were harvested, filtered (0.45-μm pore size, Millipore, SLHP033RB) and stored at −70°C in 2-ml aliquots until use. The 50% tissue culture infectious dose (TCID_50_) of each NiV pseudovirus batch was determined using a single-use aliquot from the pseudovirus bank; all stocks were used only once to avoid inconsistencies that could have resulted from repeated freezing-thawing cycles. For titration of the NiV pseudovirus, a 50-fold initial dilution was made in hexaplicate wells of 96-well culture plates followed by serial 5-fold dilutions (9 dilutions in total). The last column served as the cell control without the addition of pseudovirus. Then, the 96-well plates were seeded with trypsin-treated mammalian cells adjusted to a pre-defined concentration. After 48 h incubation in a 5% CO_2_ environment at 37°C, the culture supernatant was aspirated gently to leave 100 μl in each well; then, 100 μl of luciferase substrate (Promega, Madison, WI, USA) was added to each well. Two min after incubation at room temperature, 150 μl of lysate was transferred to white solid 96-well plates for the detection of luminescence using a Glomax microplate luminometer (Promega, Fitchburg, WI, USA). The 50% tissue culture infectious dose (TCID_50_) was calculated using the Reed–Muench method, as described previously [22].

### *In vitro* pseudovirus neutralization assay

Neutralization was measured by the reduction in *luc* gene expression, as described previously for the HIV pseudovirus neutralization assay [46]. The 50% inhibitory dilution (ID_50_) was defined as the serum dilution at which the relative light units (RLUs) were reduced by 50% compared with the virus control wells (virus + cells) after subtraction of the background RLUs in the control groups with cells only. In brief, pseudovirus was incubated with serial dilutions of the test samples (8 dilutions in a 3-fold step-wise manner) in duplicate for 1 h at 37°C, together with the virus control and cell control wells in hexaplicate. Then, freshly trypsinized cells were added to each well. Following 48 h of incubation in a 5% CO_2_ environment at 37°C, the luminescence was measured as described in the section for pseudovirus titration. The ID_50_ values were calculated with non-linear regression, i.e. log (inhibitor) vs. response (four parameters), using GraphPad Prism 6 (GraphPad Software, Inc., San Diego, CA, USA).

### Animal experiments

All mice and guinea pigs were housed and maintained in accordance with the relevant national guidelines and regulations. All procedures were carried out according to the protocols approved by the Institutional Animal Care and Use Committee of the National Institute for Food and Drug Control (NIFDC). All animals were obtained from the Institute for Laboratory Animal Resources, NIFDC. To obtain passive transferring sera, Hartley guinea pigs (females, ∼200 g in weight) were immunized three times with 200 µg of pSV1.0-NiV-F, -G, or both, followed by electroporation at an interval of two weeks. For active immunization, Balb/c mice (females, ∼15 g in weight) were inoculated with 3-fold serially diluted pSV1.0-NiV-F, -G, or both, at an initial amount of 50 µg/mouse. For the pseudovirus challenge assays, bioluminescence was detected for each mouse after anesthetization.

### *In vivo* bioluminescence imaging analysis

Bioluminescence analyses were conducted using the IVIS-Lumina III imaging system (Xenogen, Baltimore, MD, USA), as described previously [22,47]. Briefly, mice were anesthetized by IP injection of pentobarbital sodium (40 mg/kg body weight), followed by an IP injection of D-luciferin (150 μg/g body weight; Xenogen-Caliper Corp., Alameda, CA, USA). After 7 min, the bioluminescence signal was detected for each mouse with an acquisition time of 1 min. The relative bioluminescence was calculated using the photon-per-second mode with normalization for the imaging area (photons/s/cm^2^/sr) (total lux), as previously described [22].

#### Statistical analysis

Pearson’s correlation coefficient was employed to analyse the strength of the linearity between the log_10_-transformed values for the flux and NAb titres. All graphs were generated using Prism 6.0c software (GraphPad).
