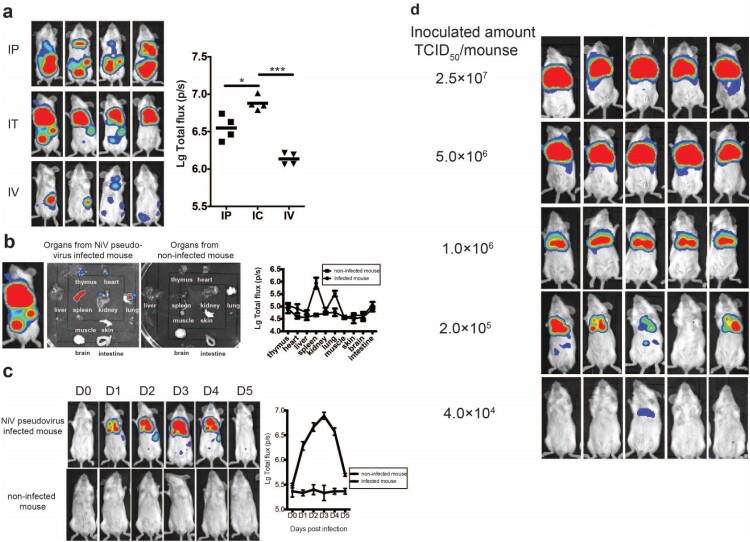# Correction

**DOI:** 10.1080/22221751.2025.2609512

**Published:** 2025-12-28

**Article title:** Nipah pseudovirus system enables evaluation of vaccines in vitro and in vivo using non-BSL-4 facilities

**Authors:** Nie J., Liu L., Wang Q., Chen R., Ning T., Liu Q., Huang W. and Wang Y.

**Journal:** *Emerging Microbes & Infections*

**Volume** 8 **Issue** 1

**DOI**: https://doi.org/10.1080/22221751.2019.1571871

When this article was first published online Figure 2 was incorrect. An inadvertent figure-assembly error in Figure 2d where the image for the 2.0×10^5^ incorrectly duplicated the 1.0×10^6^ image (marked with a red square). This figure (Fig. 2d) is intended to determine the animal infectious dose. By reviewing the original data, we found the original image of this group. The number of mice with positive detection in this dose group was also four (out of five), which is consistent with the positive-conversion rate in the misused image. Therefore, this will not affect the results of this part or the conclusions of the article.

We have thoroughly reviewed the data and confirm this error does not affect the overall conclusions of the study. However, to ensure the rigor of the manuscript and avoid potential misunderstandings, we wish to correct this figure as follows.

**Corrected Figure 2.**

**Figure:**